# Preparation of the Biodegradable Lymphatic Targeting Imaging Agent Based on the Indocyanine Green Mesoporous Silicon System

**DOI:** 10.3389/fchem.2022.847929

**Published:** 2022-02-22

**Authors:** Man Duan, Dongmei Han, Wenbin Shen, Kun Chang, Xinyu Wang, Nan Gao, Jianshi Du

**Affiliations:** ^1^ Key Laboratory of Lymphatic Surgery Jilin Province, Jilin Engineering Laboratory for Lymphatic Surgery Jilin Province, China-Japan Union Hospital of Jilin University, Changchun, China; ^2^ Department of Lymphology, Beijing Shijitan Hospital, Capital Medical University, Beijing, China; ^3^ Key Laboratory of Polyoxometalate and Reticular Material Chemistry of Ministry of Education, Faculty of Chemistry, Northeast Normal University, Changchun, China

**Keywords:** indocyanine green, lymph vessel, mesoporous silicon, LYVE-1, hyaluronic acid

## Abstract

The lymphatic system plays a crucial role in the immune system’s recognition and response to disease. Therefore, the imaging of the lymphatic system, especially lymphatic vessels, has emerged as a valuable tool for the diagnosis of metastasis. FDA-approved small-molecule dyes, namely, indocyanine green (ICG), have been widely applied to lymphatic vessels imaging. However, due to the small physical size, such molecule-based agents show no selectivity, and rapid clearance from lymph nodes. Herein, a biodegradable lymphatic targeting imaging agent based on the ICG-mesoporous silicon system (ICG@HMONs-HA) was obtained, which not only could target lymph vessels but also had a long residence time. The reported work provides a practical way for lymph vessel fluorescence imaging and paves the way for clinical translation of nanomaterial-based tracers.

## Introduction

The lymphatic system, composed of the circulatory system with the blood circulating system, plays a crucial role in the immune system’s recognition and response to disease (Oliver, 2004; Oliver and Alitalo, 2005; Tammela and Alita, 2010; Petrova and Koh, 2017). For many carcinomas, regional lymph nodes act as reservoirs where cancer cells take root and seed into other parts of the body (Bouta et al., 2018; Wang et al., 2019). Therefore, the identification of metastasis within the sentinel lymph node (SLN) is a key criterion for prognostic assessment, minimally invasive tumor staging, and treatment planning. Therefore, the imaging of the lymphatic system, especially lymphatic vessels, has emerged as a valuable tool for the diagnosis of metastasis. Currently practiced lymphatic vessels imaging techniques include positron emission tomography (PET), single photon emission computed tomography (SPECT), computed tomography (CT), and magnetic resonance imaging (MRI) (Zhang et al., 2012; Qiao et al., 2015; Polomska and Proulx, 2021). However, most of the aforementioned techniques require expensive instruments, complicated procedures, and radiocolloids, which limit their wider applications. Therefore, there is an urgent need to design non-invasive probes to accelerate the achievement of accurate SLN mapping in clinical settings (Trevaskis et al., 2015).

Fluorescence-based optical imaging is an emerging and non-invasive biomedical imaging modality and can visualize biological samples at scales from organelles, cells, tissues, and organs to small-animal whole bodies (Mellor et al., 2010; Keo et al., 2015). Compared to other imaging modalities, fluorescence imaging enables real-time, secure, and accurate visualization owing to its high sensitivity and non-radioactive nature. The FDA-approved and clinically accessible, small-molecule dyes, such as indocyanine green (ICG), have been successfully applied to SLN imaging (Kraft et al., 2018; Papayan and Akopov, 2018). However, such molecule-based agents enter both lymphatic vessels and blood capillaries with no selectivity due to their small physical size (<2 nm). Even worse, ICG displayed rapid clearance from lymph nodes after their administration, which could cause quick attenuation of fluorescence intensity and lead to false diagnostic results (Gashev et al., 2010).

Therefore, the improvement direction of ICG is to increase the targeting capability of the ICG-based imaging system to the lymphatic system. There are two main ways to increase the targeting capability. One way is to depend on the size. It was reported that the pore size of blood vessel walls is less than 6 nm (Proulx et al., 2013; Niki et al., 2015), while the pore size of lymphatic vessel walls is 30–120 nm. So, the particle size of lymphatic vessels targeting nanomaterials should be greater than 6 nm and less than 120 nm (many reports had proved that the most ideal range of the particle size of nanomaterials was between 50–100 nm) (Li et al., 2015; Trevaskis et al., 2015; Yang et al., 2017a; Rohner and Thomas, 2017), and lymphatic tracers with this size range could permeate into lymphatic capillaries after administration selectively. The other way is the specific binding. It was widely reported that lymphatic vessel endothelial receptor-1 (LYVE-1), which is overexpressed on lymphatic endothelium, and hyaluronic acid (HA) acted as the substrate for LYVE-1 (McElroy et al., 2009; Yang et al., 2017b). The strong binding affinity between LYVE-1 and HA-coated ICG-based lymphatic tracers would increase the retention time of tracers in the lymph vessel and offered an optimal time window for fluorescence imaging.

Ongoing research efforts in material chemistry facilitate the development of novel nanomaterials-based lymphatic tracers with tunable size, which accelerates the application of fluorescence imaging in real-time identification of lymph vessel. Moreover, the nanomaterials protect the tracers from premature release and prolonged the circulation time in the lymphatic system. Up to now, a wide range of nanomaterials based on inorganic, organic, and multifunctional nanoprobes have been reported, and applied for lymph vessel mapping (Kitai et al., 2005; Unno et al., 2007; Sharma et al., 2013; Liu et al., 2014; Liang et al., 2015; Sugiura et al., 2015; Oladipo et al., 2017; Rockson, 2017; Shi et al., 2018; Wu et al., 2020; Jin et al., 2021; Wang et al., 2021; Zhao et al., 2021). Unfortunately, non-degradable nanomaterials could accumulate into the cells and organs, and exert the damage effect. Thus, for potential clinical application, it is necessary that nanotracers could be completely eliminated from the body after mapping as soon as possible.

Herein, a biodegradable hollow mesoporous organo-silica nanoparticle (HMON) was chosen as the carrier with the average particle size of 90 nm. This nanoparticle could specifically permeate into the lymphatic system, due to its appropriate size (Xie et al., 2009; Yukuyama et al., 2020). Most importantly, this nanoparticle would degrade gradually over a period of 7 days, after which almost no intact particles remain. The resultant biodegraded nanoparticle has reduced *in vivo* toxicity compared to the reported non-degradable ones.

**SCHEME 1:**
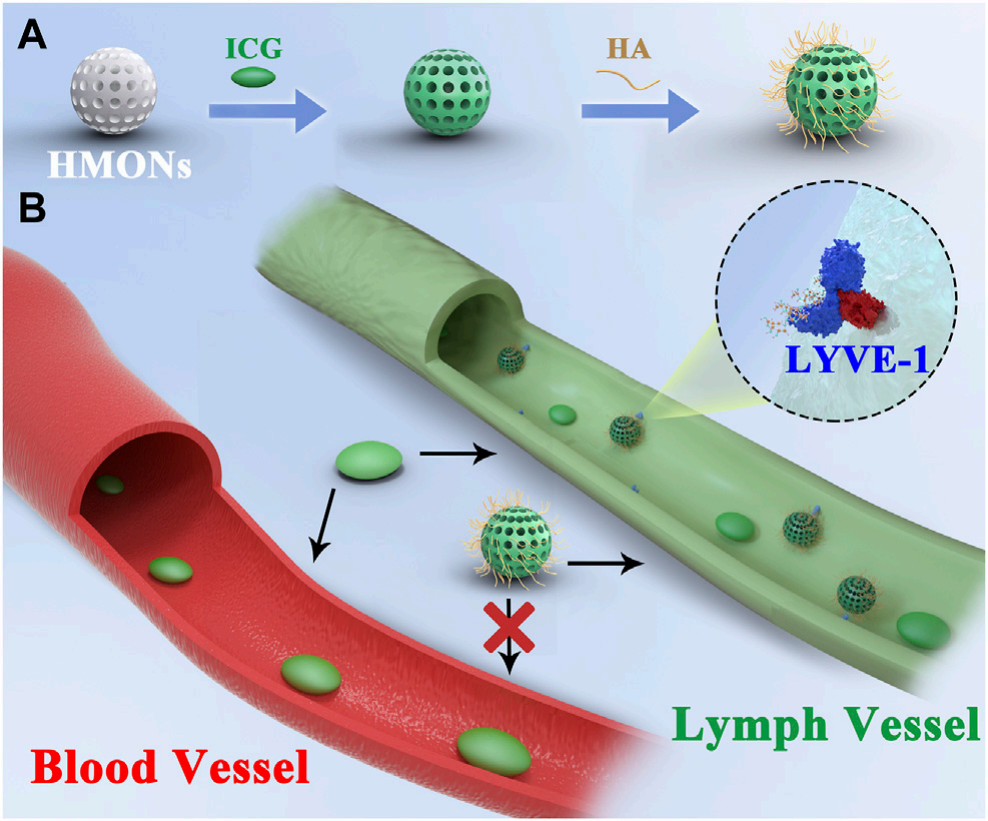
**(A)** Synthesis process of ICG@HMONs-HA. **(B)** Schematic of lymph vessel targeting fluorescence imaging of ICG@HMONs-HA.

Then, ICG and HA were modified on the surface of the biodegradable HMON (named ICG@HMONs-HA), and the strong binding affinity between LYVE-1 and HA would increase the imaging time (Scheme 1). The reported work provides a practical way for lymph vessel fluorescence imaging and paves the way for the clinical translation of nanomaterial-based tracers.

## Results and Discussion

Commercial aminated self-degradable organic mesoporous silicon (HMONs-NH_2_) with an average particle size of 90 nm was used to determine the amount of primary amine exposed on the surface of mesoporous silicon by titration, and the determination result was about 0.6 μmol primary amine per mg mesoporous silicon. Therefore, in this experiment, each modification reaction took 5 mg mesoporous silicon as a unit; that is, a unit of mesoporous silicon contains about 3 μmol exposed primary amine.

**FIGURE 1:**
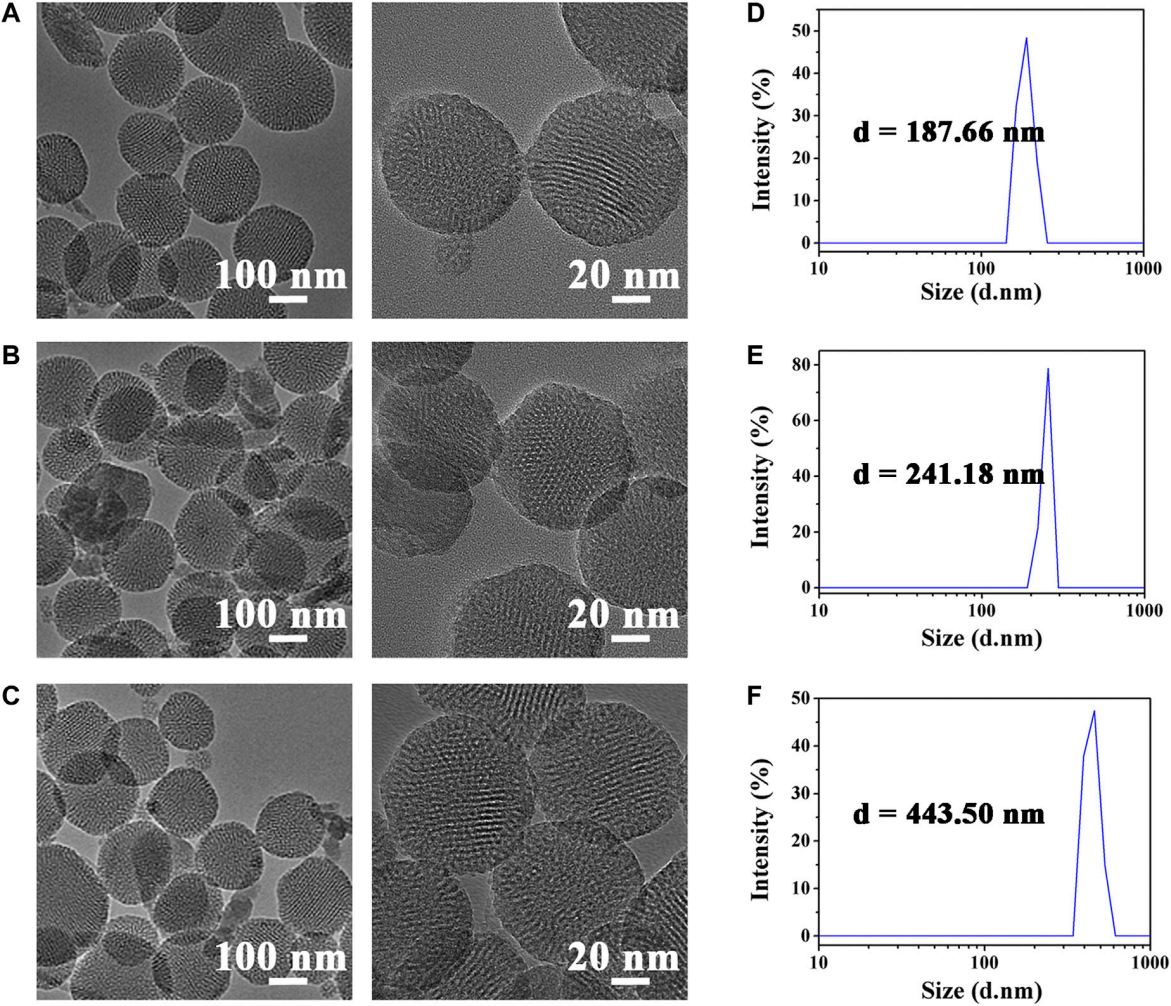
TEM images of HMONs-NH2 **(A)**, ICG@HMONs **(B)**, and ICG@HMONs-HA **(C)**. Average hydrodynamic diameter of HMONs-NH2 **(D)**, ICG@HMONs **(E)**, and ICG@HMONs-HA **(F)** determined by DLS.

Before *in vivo* fluorescence imaging experiments, we first characterized the physical properties of HMONs-NH_2_. The morphology of HMONs-NH_2_ under the anhydrous state was imaged using transmission electron microscopy (TEM). As shown in Figure 1, the TEM images revealed that HMONs-NH_2_ has a uniform and monodispersed spherical shape. It is also observed that HMONs-NH_2_ had a diameter of 100 nm, which is ideal for lymphatic tracing because nanotracers with this size will specifically permeate into lymphatic capillaries (Figure 1). This result is further confirmed by the particle size distribution measured using dynamic light scattering (DLS). The characterization experiments previously showed that the HMONs-NH_2_ suspension injection used in the clinic possesses favorable physical properties, such as tunable size and optical absorbance, making it an excellent nanocarrier for lymphatic vessels imaging. Then, ICG-COOH was modified on HMONs-NH_2_ using carboxyl-amino reaction. After the reaction, the amount of the residual amino group was determined by titration, and the amount of modified ICG was calculated. Titration results showed that there were 1.9 μmol exposed primary amine left per 5 mg mesoporous silicon, which means there were 1.1 μmol ICG modified on per 5 mg HMONs-NH_2_.

To facilitate target delivery and increase the retention time of tracers in the lymphatic vessels, the ICG@HMONs nanoparticles were next coated with hyaluronic acid (HA) to form ICG@HMONs-HA. The successful deposition of HA was confirmed by TEM images, DLS, and zeta-potential measurements. The average hydrodynamic diameter of HMONs-NH_2_ was about 188 nm, whereas the diameter of ICG@HMONs-HA increased to 443 nm after HA deposition. The structure of ICG@HMONs-HA was determined by FT-IR (Supplementary Figure S1) and UV–Vis (Supplementary Figure S2). Also, the data of zeta-potential were decreased from 13.49 ± 2.21 mV of HMONs-NH_2_ to −7.19 ± 3.37 mV of ICG@HMONs-HA (Supplementary Figure S3). The previous results indicated that HA has also been modified on the surface of ICG@HMONs. Therefore, the HA deposition on the surface of HMONs could avoid premature ICG leakage and realize lymphatic vessels-specific accumulation.

**FIGURE 2:**
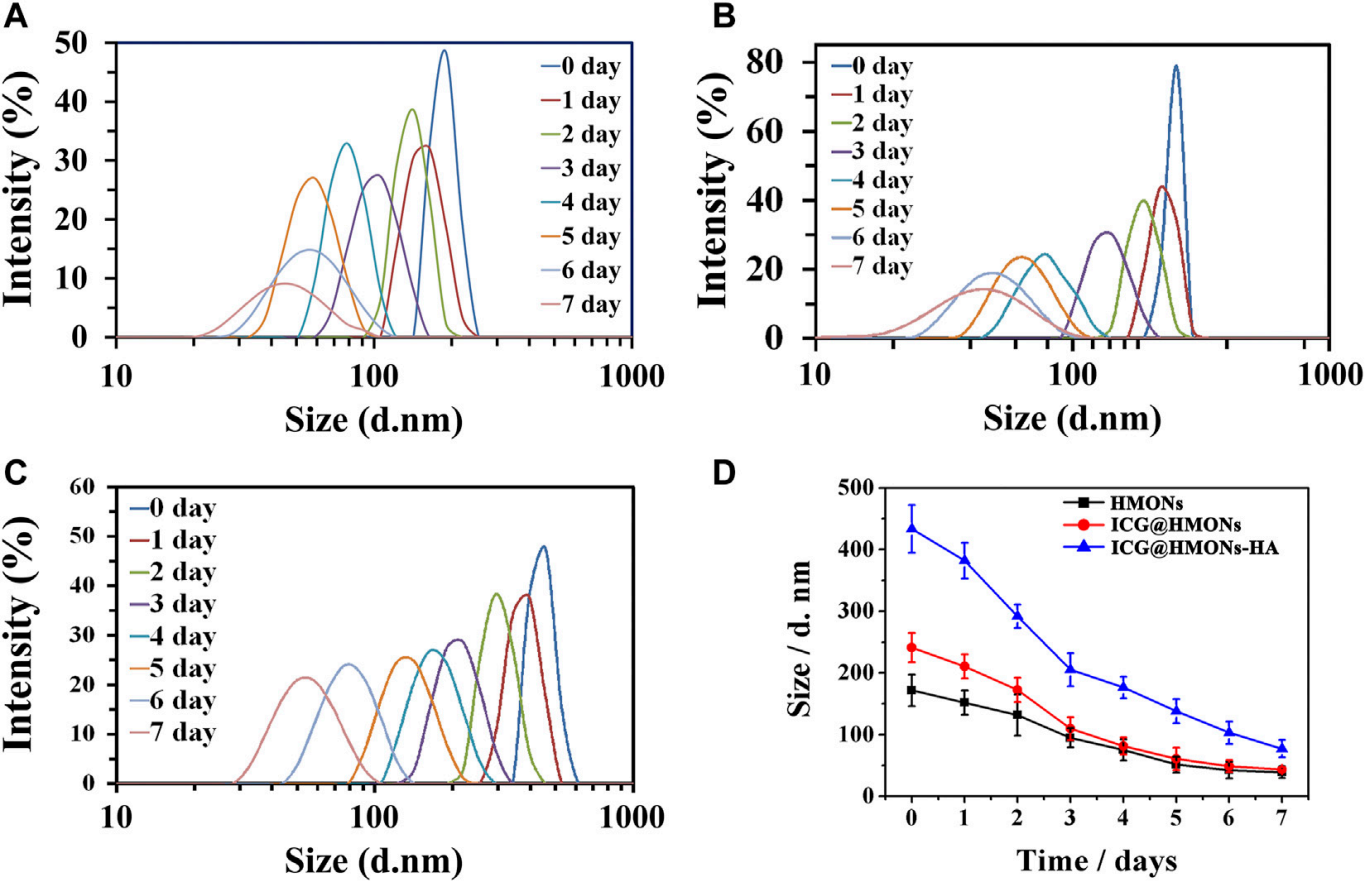
Change of hydrodynamic diameter of HMONs-NH_2_ **(A)**, ICG@HMONs **(B)**, and ICG@HMONs-HA **(C)** in 7 days determined by DLS. **(D)** Summary of the change of hydrodynamic diameter for the three nanomaterials. Each experiment had been repeated three times. Error bars indicate ±*s*.d.

HMONs-NH_2_ developed in the present study was assessed for its degradability under simulated physiological conditions (pH 7.4, 10 mM PBS buffer). HMONs-NH_2_, ICG@HMONs, and ICG@HMONs-HA could all be completely degraded under physiological conditions in 7 days incubation, as demonstrated by microscopic observation and by DLS measurements (Figure 2; Supplementary Figure S4). These phenomena proved that ICG@HMONs-HA was biodegradable under physiological conditions. The successful fabrication of biodegradable mesoporous silica nanoparticles paved the way for the application in lymphatic vessels imaging.

First, the potential toxicity of ICG@HMONs-HA was evaluated by the MTT assay. No apparent cytotoxicity was observed when the ICG@HMONs-HA concentration was as high as 2.0 mg/ml (Supplementary Figure S5). The toxicities of ICG@HMONs-HA compared to some commercial nanoparticles were also investigated (Supplementary Figure S6). The fluorescence imaging properties of ICG@HMONs-HA were investigated in various cell types, including mouse lymph: normal lymphatic endothelial cells (MLEC), murine macrophage (RAW 264.7) cells, and mouse colon cancer cells (CT26). The reasons for selection: RAW264.7 is a normal mouse cell and does not contain the LYVE-1 receptor; CT26 is a mouse cancer cell containing a small amount of the LYVE-1 receptor; and MLEC is a mouse lymphatic endothelial cell and contains a large number of LYVE-1 receptors.

The three kinds of cells were placed in a 24-well plate with a 1 cm x1 cm glass sheet, and the culture medium was added, respectively, for culture so that the cells grew on the glass sheet. The cells were stained with ICG or ICG@HMONs-HA, and then the glass sheets were washed with sterile normal saline for 1, 2, and 3 or 5 times. (One-time cleaning was completed by soaking all the glass sheets grown on the cell wall in sterile normal saline, standing for 5 min and taking them out.) The cells were fixed with 4% paraformaldehyde and imaged by using the confocal laser scanning microscope.

**FIGURE 3:**
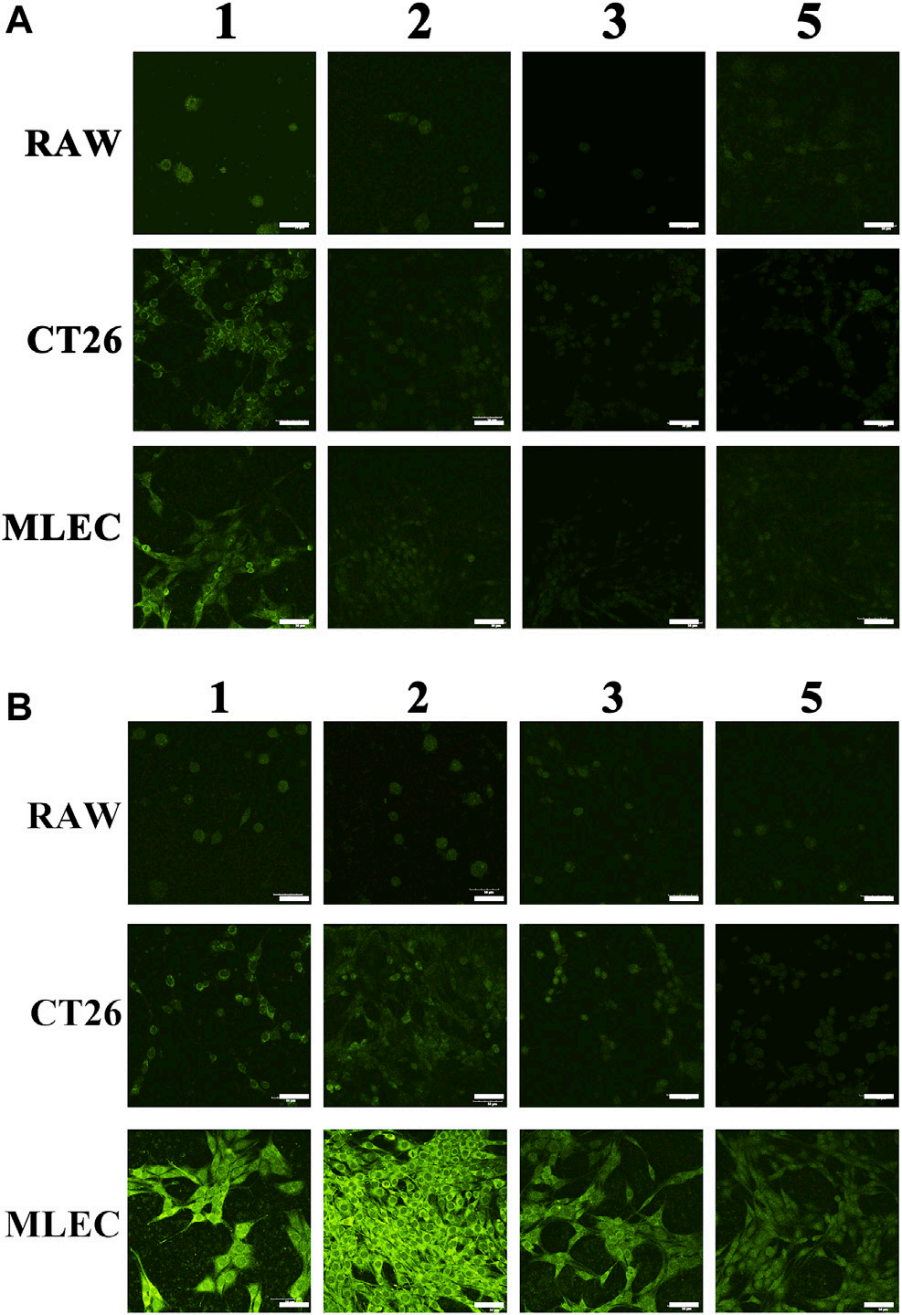
Staining effect of ICG **(A)** or ICG@HMONs-HA **(B)** on three kinds of cells. The numbers 1, 2, 3, and 5 in the picture represent the cleaning of cells for 1, 2, 3, or 5 times after staining. The scale bars equal 50 μm.

Figure 3A shows the staining effect of ICG on three kinds of cells. Figure 3B shows the staining effect of three kinds of cells using ICG@HMONs-HA. The numbers 1, 2, 3, and 5 in the picture represent the cleaning of cells for 1, 2, and 3 or 5 times after staining. It can be concluded that the use of ICG staining is neither selective nor firm in binding to cells (easy to be metabolized). Using ICG@HMONs-HA staining, both selectivity (specific binding to MLEC) and firm binding to cells (not easy to be metabolized) could be observed (Supplementary Figure S7). In conclusion, the strong binding affinity between HA and LYVE-1 could facilitate the migration and retention of ICG@HMONs-HA for effective lymphatic vessels imaging.

**FIGURE 4:**
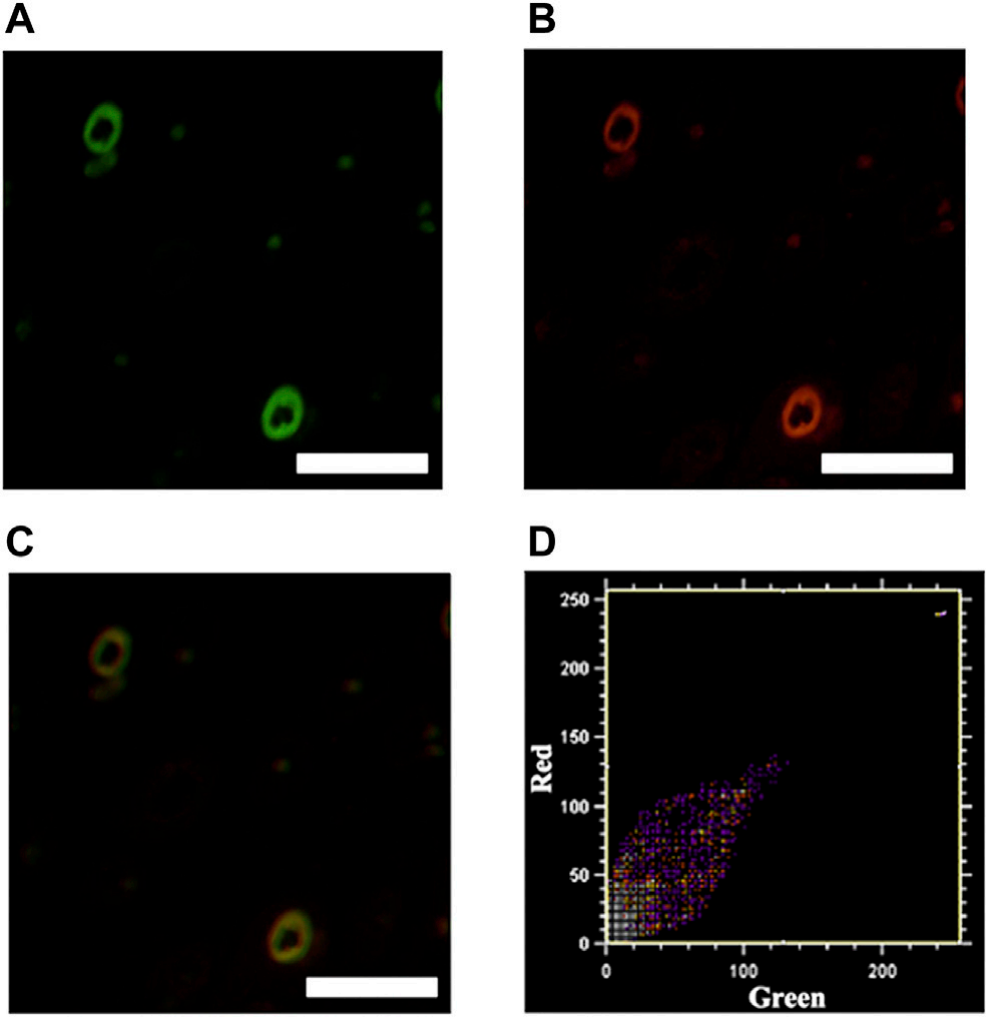
Mouse tissue sections stained with ICG@HMONs-HA **(A)** and LYVE-1 antibody **(B)**, and merged image **(C)**. Scale bars equal 200 μm. **(D)** Co-localization analysis using Co-localization Finder plugins in Fiji ImageJ software.

To further demonstrate the specific binding of ICG@HMONs-HA to LYVE-1, mouse leg tissues were sectioned, sliced, and stained with ICG@HMONs-HA and LYVE-1 antibodies. As shown in Figure 4, colocalization of LYVE-1 and ICG@HMONs-HA can be recognized on the overlaid images, indicating the binding of ICG@HMONs-HA to LYVE-1 within the lymphatic vessels, and Pearson’s colocalization coefficient was 0.9577 (Supplementary Table S1).

## Materials and Methods

### Materials

Commercial aminated self-degradable organic mesoporous silicon (HMONs-NH2) with an average particle size of 90 nm was purchased from So-Fe Biomedical Technology Co., Ltd (Shanghai, China). ICG-COOH was purchased from Xinqiao Biotechnology Co., Ltd (Hangzhou, China). ICG was provided by China-Japan Union Hospital of Jilin University. HA was purchased from Aladdin Biochemical Technology Co., Ltd (Shanghai, China). All cell-culture used reagents were purchased from Gibco. Other chemicals and solvents were purchased from commercial suppliers and used without further purification. All reactions were performed under a purified nitrogen atmosphere.

### Titration Assay

The principle is that the reaction between primary amine and salicylaldehyde can produce Schiff base and water. Then the excess salicylaldehyde is titrated with pyridine standard solution of sodium methoxide to calculate the amount of salicylaldehyde consumed by the primary amino group, and then the content of the primary amino group is calculated.

### Synthesis of ICG@HMONs-HA

1) 1 mg of ICG-COOH (about 1.4 μmol), 1.44 mg dicyclohexylcarbodiimide (DCC) (about 7.0 μmol), and 0.8 mg N-hydroxysuccinimide (NHS) (about 7.0 μmol) are taken. The mixture is then dissolved in 1 ml of N,N-dimethylformamide (DMF) and stirred at room temperature for 24 h to obtain carboxyl activated ICG.
2) 5 mg of HMONs-NH2 is taken and dispersed in 2 ml DMF, and the reaction products of step (1) are added directly; stirring is continued at room temperature for 24 h, then centrifuged at 10,000 rpm/min to collect the precipitation, and the precipitation is washed with DMF three times to obtain ICG-modified mesoporous silicon (ICG@HMONs) (at this time, about half of the amino groups on the surface of HMONs have been reacted, and half of the amino groups remain).
3) 5 mg of commercially available hyaluronic acid (abbreviated as HA, derived from Streptococcus equi) (compared to the other half of the amino groups remaining on the surface of mesoporous silicon, the amount of hyaluronic acid is in large excess so that all amino groups on the surface of mesoporous silicon can be completely reacted in the next step) is taken; 0.103 g DCC (about 0.5 mmol, large excess) and 0.0575 g NHS (about 0.5 mmol, large excess) were dissolved in 5 ml water and stirred at room temperature for 24 h to obtain carboxyl activated HA.
4) ICG@HMONs obtained in step (2) was dispersed in 5 ml methanol, then the reaction product in step (3) was directly added, stirred at room temperature for 8 h, then centrifuged at 10,000 rpm/min to collect the precipitation, washed with DMF for three times, and washed with water for three times to obtain the final ICG@HMONs-HA nanotracers.

### Biodegradation Assay

The biodegradation assay of HMONs was carried out under the condition of the simulated physiological environment. HMONs (50 mg) were dispersed in the PBS buffer (10 ml), which was placed in a dialysis bag (MW = 3.5 kDa). The decomposed HMONs were taken out from a dialysis bag after 7 days incubation, and the collected suspension was dried under vacuum for the following TEM and DLS characterizations. The size analysis was taken by Zetasizer Nano (Malvern Instruments, Worchestershire, United Kingdom) at room temperature. Triplicate measurements were carried out. The data were plotted as scattered light intensity *vs.* diameter.

### Cellular Accumulation and Quantification of the Fluorescence Intensity

MLEC cells, RAW 264.7 cells, and CT26 cells were seeded on six-well plates at 1 × 10^5^ cells per well. After 12 h incubation, cells were treated with ICG or ICG@HMONs-HA. Then cells were washed different times with PBS and incubated in a fresh medium. The fluorescences of cells were estimated by CLSM (FV3000, Olympus Corporation).

## Conclusion

In summary, we validated that ICG@HMONs-HA is an effective imaging agent for lymphatic vessels mapping due to its specific lymphatic vessels accumulation and long retention time. HMONs-based nanotracers could non-invasively identify the lymphatic vessels at high sensitivity *in vivo*. There are several advantages to use ICG@HMONs-HA as the lymphatic vessels mapping agent. The most prominent one is that they have an average diameter of 90 nm, an ideal size allowing them to differentiate the lymphatic vessels from the blood vessel. This would also provide much high-quality image guidance and reduce the number of false-negative detections. Second, the biodegraded HMONs coated with endogenous HA have reduced *in vivo* toxicity compared to the reported non-degradable ones. More importantly, the binding of HA to LYVE-1 receptors on the lymphatic endothelial cells could further facilitate the retention of ICG@HMONs-HA within the lymphatic vessels. The reported work provides a practical way for lymphatic vessels selective identification and paves the way for clinical translation of biodegradable nanotracers.

## Data Availability

The original contributions presented in the study are included in the article/Supplementary Material, further inquiries can be directed to the corresponding authors.
