# Supplementary material for: Preparation of the Biodegradable Lymphatic Targeting Imaging Agent Based on the Indocyanine Green Mesoporous Silicon System
Source: Front Chem. 2022 Feb 22;10:847929. doi: 10.3389/fchem.2022.847929 (PMC8902163; doi:10.3389/fchem.2022.847929)
Supplement: Supplementary file 1 [file DataSheet1.docx]

Supplementary Material

# Experimental Procedures

**Materials.** Commercial aminated self-degradable organic mesoporous silicon (HMONs-NH_2_) with an average particle size of 90 nm was purchased from So-Fe Biomedical Technology Co., Ltd (Shanghai, China). ICG-COOH was purchased from Xinqiao Biotechnology Co., Ltd (Hangzhou, China). ICG was provided by China-Japan Union Hospital of Jilin University. HA was purchased from Aladdin Biochemical Technology Co., Ltd (Shanghai, China). All cell-culture used reagents were purchased from Gibco. Other chemicals and solvents were purchased from commercial suppliers and used without further purification. All reactions were performed under a purified nitrogen atmosphere.

**TEM images:** 10 μL aliquots of the samples were dropped on 300 mesh carbon-coated copper grids for 10 min. The remaining solution was removed and air dried. The images were taken by a FEI Tecnai G2S-Twin operated at a voltage of 200 kV.

**Cell Culture:** MLEC cells, obtained from Procell Life Science&Technology Co.,Ltd. (Wuhan, China), were grown on plates in MEM media containing 10% fetal bovine serum, 1% serum L-glutamate and 1% streptomycin in a humidified atmosphere of 5% CO_2_ and 95% air. The media were changed every three days, and the cells were passaged by trypsinization before confluence.

**Cytotoxicity Assays:** Cell viability was measured by 3-(4,5-dimethylthiazol-2-yl)-2,5- diphenyltetrazoliumbromide (MTT) assays. MLEC cells were seeded at a density of 5000 cells/well on 96-well plates for 24 h. Cells were incubated for 24 h and were treated with the samples with different concentration and then MTT assays were carried out. To determine toxicity, 10 μL of MTT solution was added to each well and the cells were incubated for 4 h. After adding 100 μL DMSO, the absorbance of formazan was read at 490 nm on a SpectraMax M5 microplate reader. Three replicates were done for each treatment group.

**Cell Imaging:** ICG or ICG@HMONs-HA was coincubated with preseeded MLEC cells in 24-well plates for fluorescence microscopy analysis experiments. After 24 h incubation, old media were removed and cells were washed three times with PBS. The confocal laser scanning microscopy (FV3000, Olympus Corporation) was employed to image the cellular distribution of ICG or ICG@HMONs-HA.

**Mouse tissue section.** All animal studies were conducted in accordance with the principles and procedures outlined in the Guide for the Care and Use of Laboratory Animals and were approved by the Institutional Animal Care and Use Committee of Jilin University. After sacrificed, mouse leg tissues were harvested and were sectioned into 10 μm slices. Slices were incubated with rat anti-mouse LYVE-1 antibody (modified with Rhodamine B) and ICG@HMONs-HA (1 mg/ml) at room temperature for 2 h. Between each step, the slices were gently washed 5 times with 20 mM PBS solution for 5 min each time. The staining observation was done with the confocal laser scanning microscopy (FV3000, Olympus Corporation).

# Supplementary Figures and Tables





**Fig. S1.** FT-IR spectra of HMONs (black), ICG (red), HA (blue) and ICG@HMONs-HA (green).


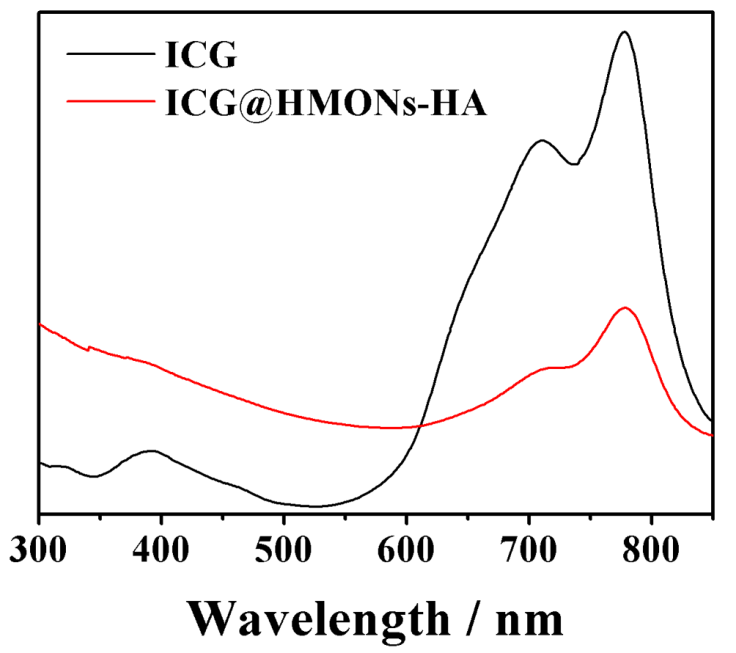


**Fig. S2.** UV-Vis spectra of ICG (black) and ICG@HMONs-HA (red).


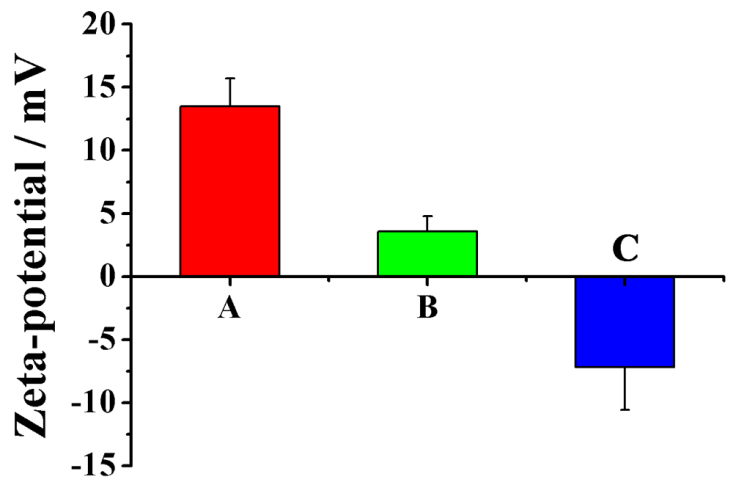


**Fig. S3** Zeta-potential of HMONs-NH_2_ (A), ICG@HMONs (B) and ICG@HMONs-HA (C). Each experiment had been repeated three times. Error bars indicate ±s.d.


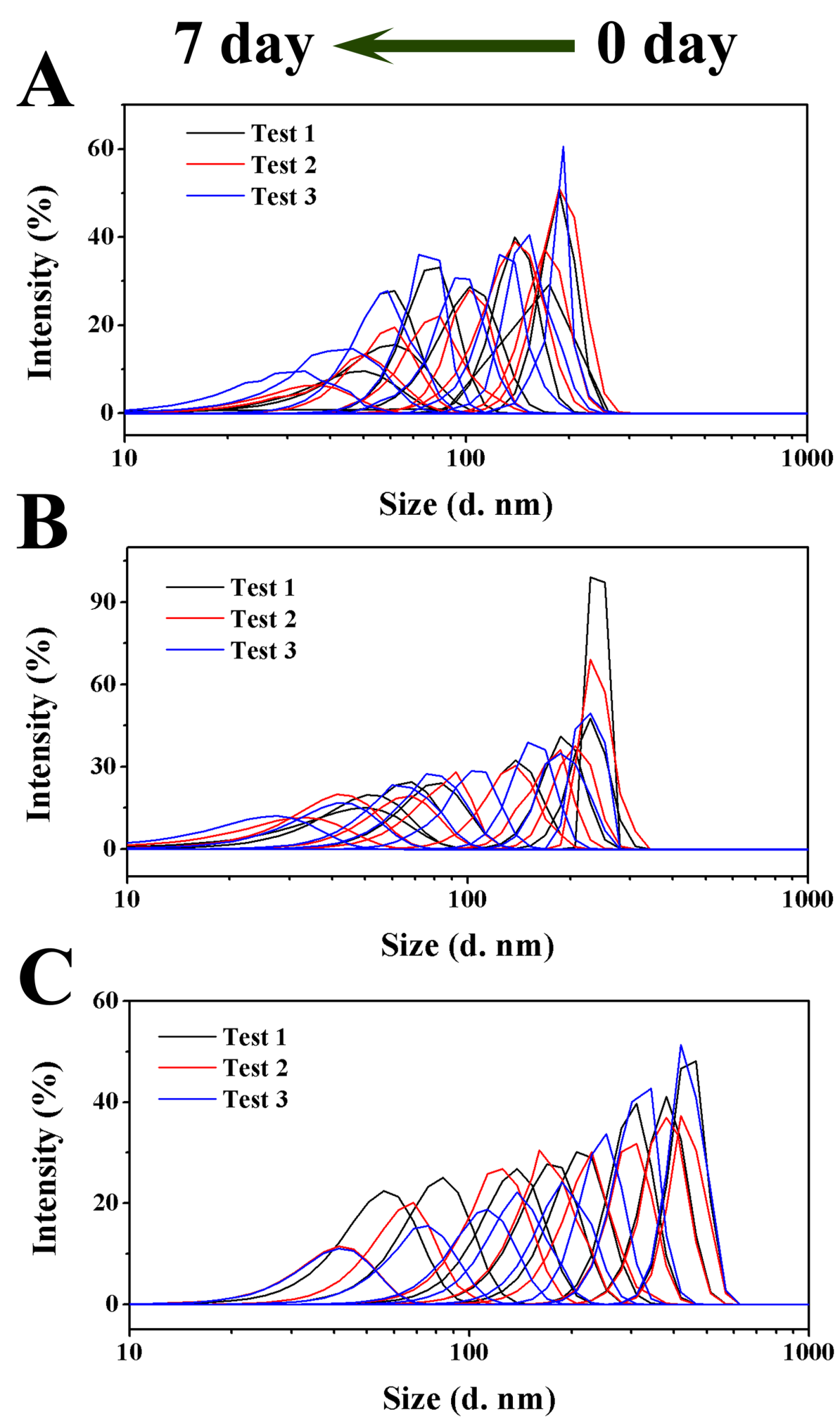


**Fig. S4** The change of hydrodynamic diameter of HMONs-NH_2_ (A), ICG@HMONs (B) and ICG@HMONs-HA (C) in 7 days determined by DLS. Each experiment had been repeated three times


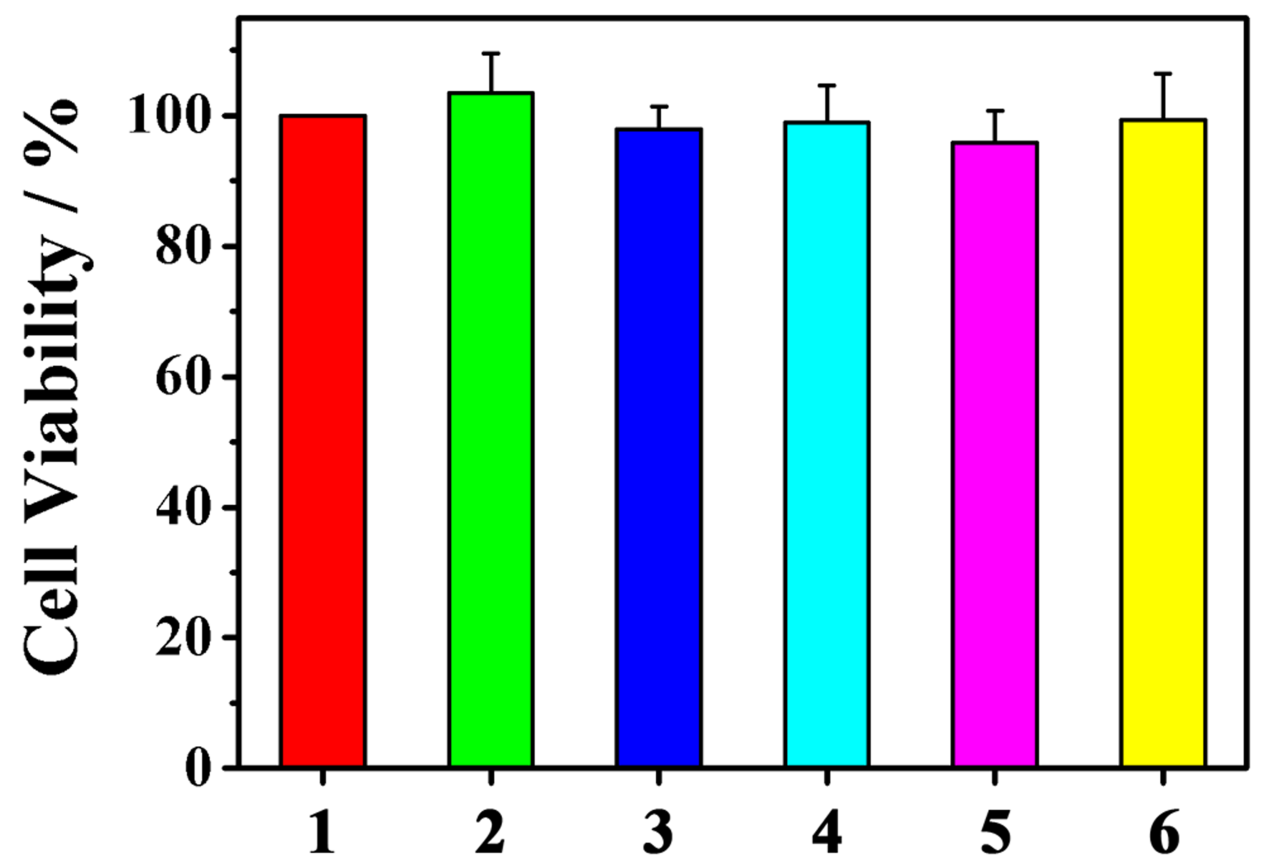


**Fig. S5** The cell toxicity of different concentrations of ICG@HMONs-HA co-incubated with RAW cells for 24 h. Each experiment had been repeated three times. Error bars indicate ±s.d. 1: control (RAW cells only); 2: add 0.1 mg/ml ICG@HMONs-HA; 3: add 0.3 mg/ml ICG@HMONs-HA; 4: add 0.5 mg/ml ICG@HMONs-HA; 5: add 1.0 mg/ml ICG@HMONs-HA; 6: add 2.0 mg/ml ICG@HMONs-HA.

.
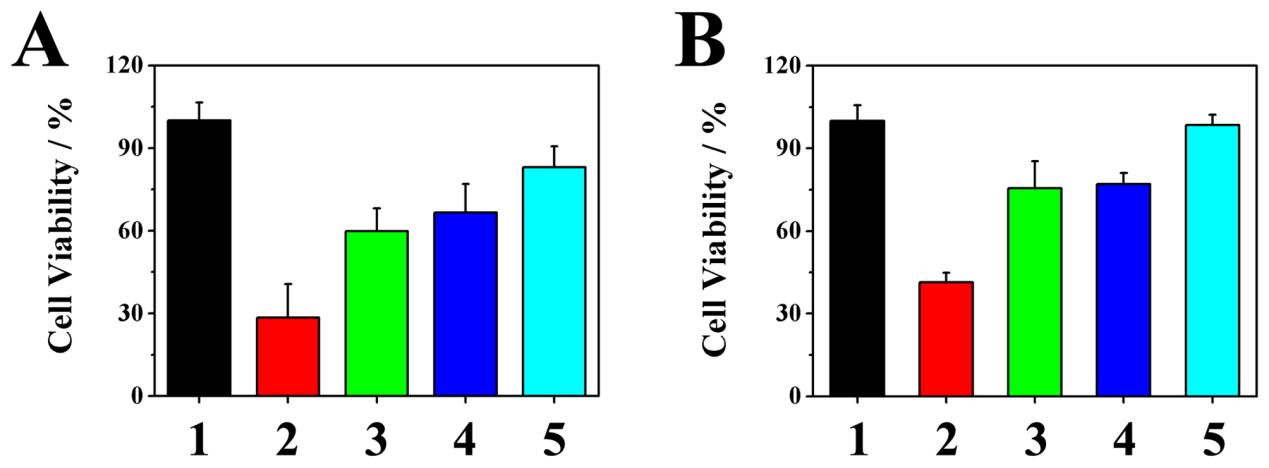


**Fig. S6** Effects of different nanoparticles on cytotoxicity by using MTT assay with MLEC cells. Each experiment had been repeated three times. Error bars indicate ±s.d. 1: Control (MLEC cells only); 2: commercial Au nanoparticle (100 nm); 3: commercial carbon nanotubes (100 nm); 4: commercial SiO2 nanoparticle (100 nm); 5: ICG@HMONs-HA. (A) Nanoparticles were incubated with MLEC cells for 7 days, and then measured the cell viability by MTT. (B) Nanoparticles were first incubated under physiological conditions (10mM Tris, pH 7.4, 37oC) for 7 days, then incubated with MLEC for additional 24 hours, and detected the cell viability by MTT. These results showed that due to the aggregation effect of nanoparticles, prolonged incubation or placement would lead to obvious cytotoxicity. However, due to the biodegradability of ICG@HMONs-HA, prolonged incubation or placement does not cause significant cytotoxicity.


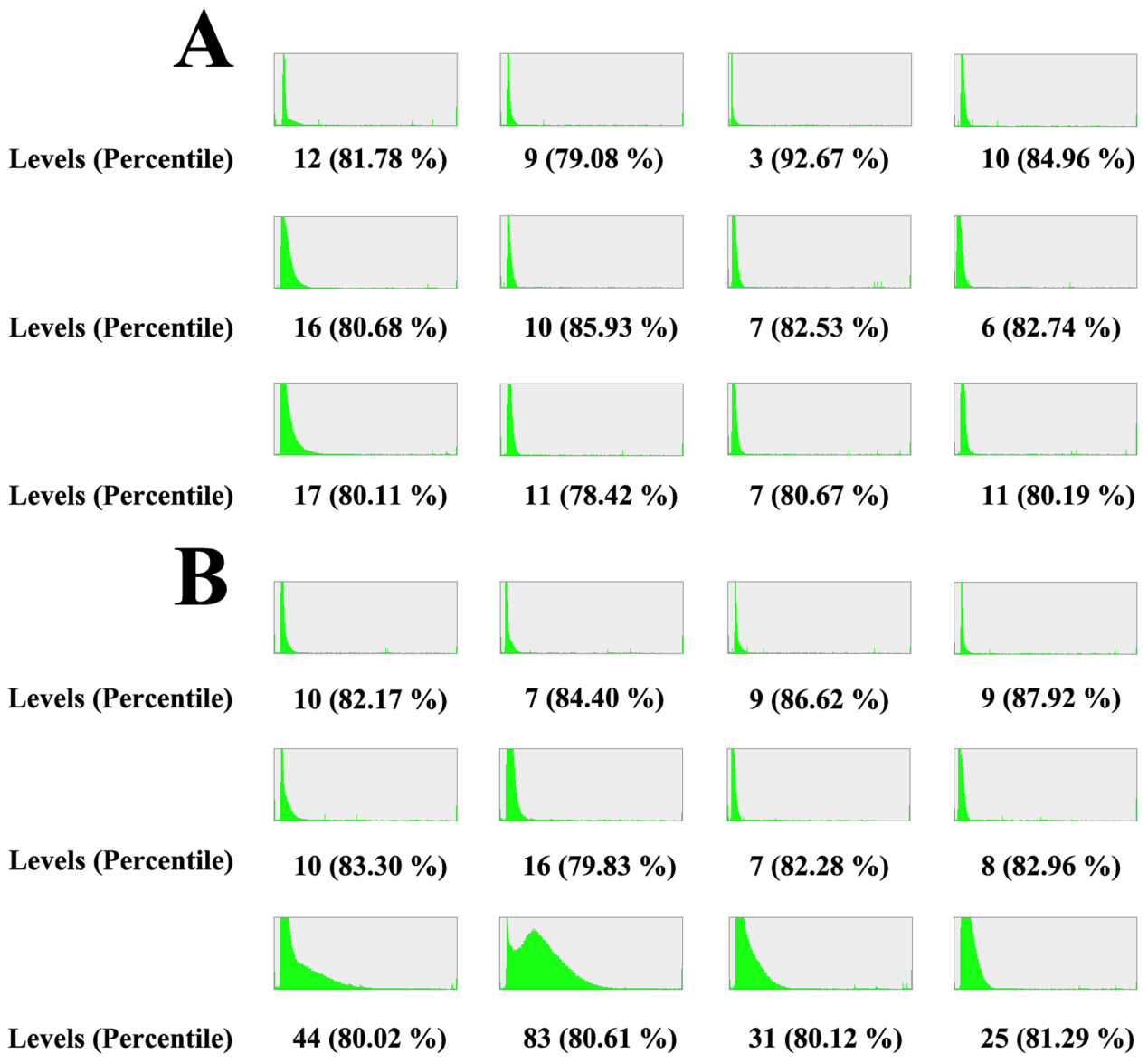


**Fig. S7** The levels values of the green fluorescence channel were evaluated around the 80% percentile using Adobe Photoshop CS3 software.

**Table S1**. The Co-location coefficients obtained by Colocalization Finder plugins in Fiji Image J software.

| Pearson's_Rr | Overlap_R | k1 | k2 |
| --- | --- | --- | --- |
| 0.9576826 | 0.9563791 | 1.013518 | 0.902461 |
